# Delayed recognition of pediatric calcaneal osteomyelitis: a case report

**DOI:** 10.1186/s13256-015-0657-x

**Published:** 2015-09-02

**Authors:** Alvin James Mallia, Neil Ashwood, Georgios Arealis, Frank Bindi, Georgiana Zamfir, Ilias Galanopoulos

**Affiliations:** Department of Trauma and Orthopaedics, Queens Hospital Burton, Burton-on-Trent, UK

**Keywords:** osteomyelitis, calcaneal, paediatrics, Orthopaedics, Sever’s

## Abstract

**Introduction:**

The diagnosis of calcaneal osteomyelitis is a challenge, and diagnostic delays have been reported in the literature. The progression is often indolent, laboratory results commonly fail to reveal an underlying infectious process and radiographs changes are seen after 7 days. We discuss the literature on the diagnosis and treatment of calcaneal osteomyelitis which can result in long-term sequelae in the pediatric patient.

**Case presentation:**

A 9-year-old white boy presented to our institution with heel pain and an inability to weight bear. There was a 10-day delay in diagnosis of calcaneal osteomyelitis, with a total of three presentations to our emergency department. The condition was misdiagnosed as Sever’s disease on two separate occasions with discharge home. On his third presentation the diagnosis was finally clinched when he developed more definitive signs and symptoms, with pyrexia and signs of lymphangitis. Magnetic resonance imaging revealed diffuse osteomyelitis of his calcaneum. He underwent surgery and 2 weeks of antibiotics administered intravenously, followed by 4 weeks of oral therapy. We are happy to report a good recovery without any complications at his 12-month follow up.

**Conclusions:**

Physicians should include calcaneal osteomyelitis as a differential in any child presenting with heel pain. Delays in the diagnosis can result in disastrous complications in the pediatric patient, such as growth arrest.

## Introduction

Calcaneal osteomyelitis is often a diagnostic challenge to the clinician. The condition distinguishes itself from long bone osteomyelitis with cases exhibiting less impressive signs and symptoms. Blood results are frequently marginal, and initial plain radiographs are often normal. The lengthy differential diagnosis of heel pain in the pediatric patient serves to only further delay the diagnosis. Delays in initiating treatment can result in disastrous complications in the pediatric patient, such as growth disturbances, chronic osteomyelitis and spread to adjacent joints. This case report aims to highlight the challenges of diagnosing calcaneal osteomyelitis, and in light of the dire consequences of delayed diagnosis, the importance of its consideration in all children presenting with heel pain.

## Case presentation

A 9-year-old white boy from the United Kingdom presented to our emergency department generally unwell, and unable to weight-bear due to a painful swelling of his left foot, with no preceding history of trauma. Ten days prior to this admission he woke from bed unable to walk on his left foot. Two days later he attended the emergency department, feeling otherwise well in himself. He received a clinical diagnosis of Sever’s disease and was discharged to have physiotherapy in the community.

**Fig. 1:**
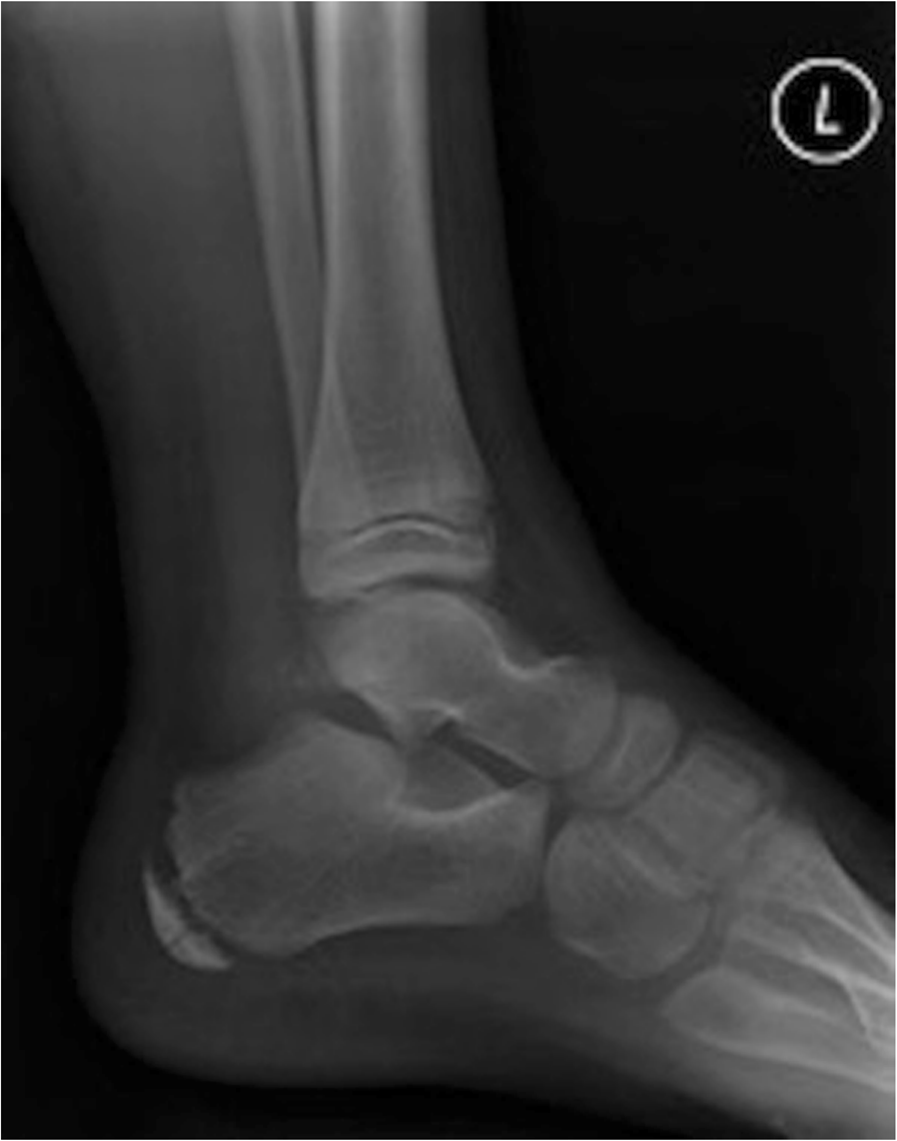
Soft tissue swelling and tenderness is noted to the calcaneum. Modified technique has been applied. The calcaneal apophysis appears sclerosed and fragmented, but this can be a normal appearance in the developing calcaneum. There is suggestion of some widening of the talocalcaneal joint medially which may correlate with appearances noted at the subtalar joints on the ankle projections

**Fig. 2:**
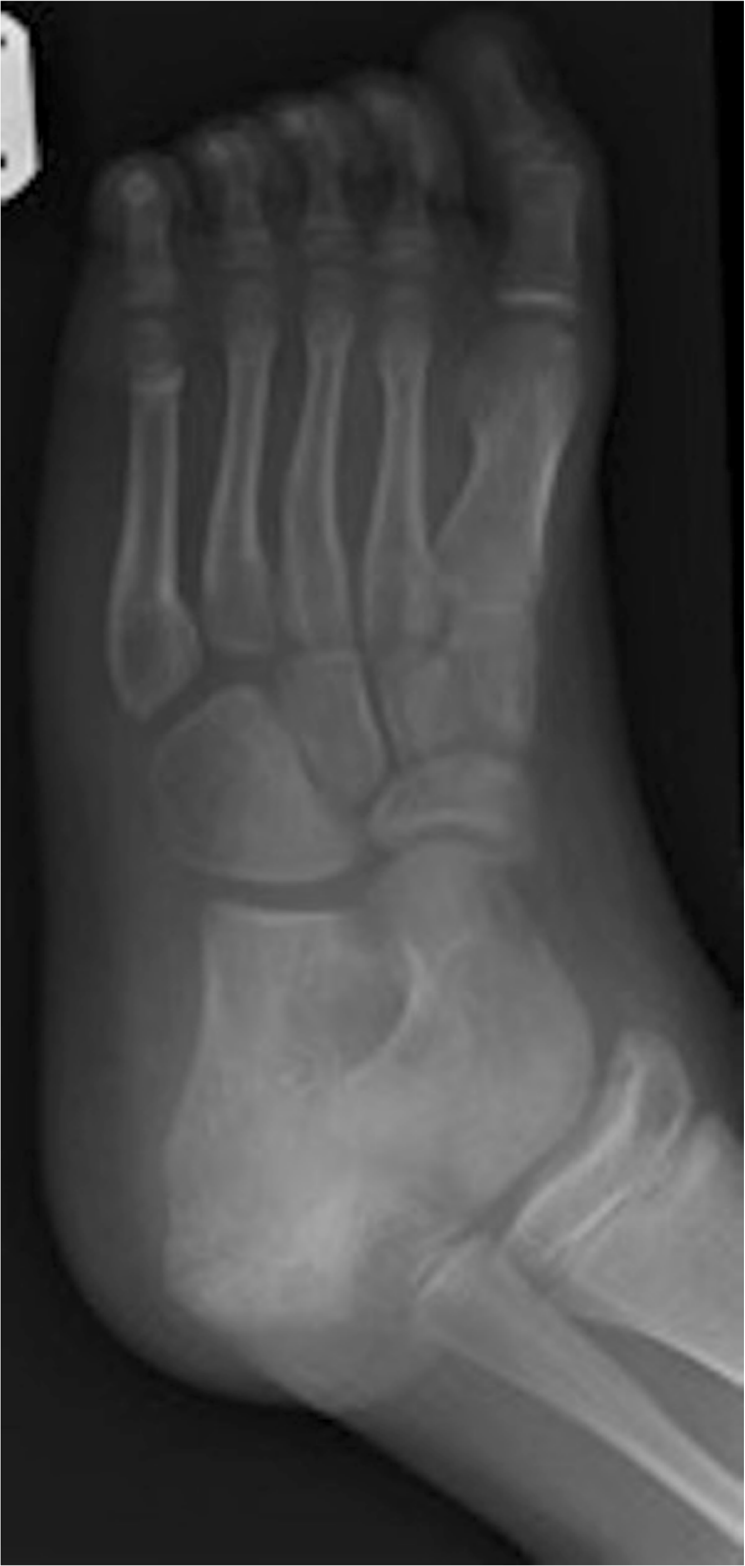
Difficult examination due to patient distress. The foot and ankle has been held in a moderate degree of plantar flexion and internal rotation. Soft tissue swelling and posterior effusion is noted. Allowing even for the immature skeleton and positioning there appears to be significant widening of the subtalar joints and as this is the specific area of tenderness, bruising, inflammation, and some subtalar subluxation is suspected

Four days after the first discharge from accident and emergency, his foot became increasingly painful so he returned to the hospital. This time clinical findings included a painful, erythematous, hot swollen heel and ankle. Plain radiographs were obtained with increased density noted within the calcaneal apophysis (Figs. 1 and 2). He was again discharged with a diagnosis of Sever’s disease. The features of Sever’s disease, however, are typically bilateral without the erythema and heel swelling.

**Fig. 3:**
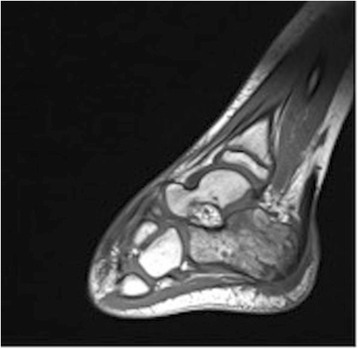
Sagittal magnetic resonance imaging scan of the left foot revealing osteomyelitis of the calcaneum

**Fig. 4:**
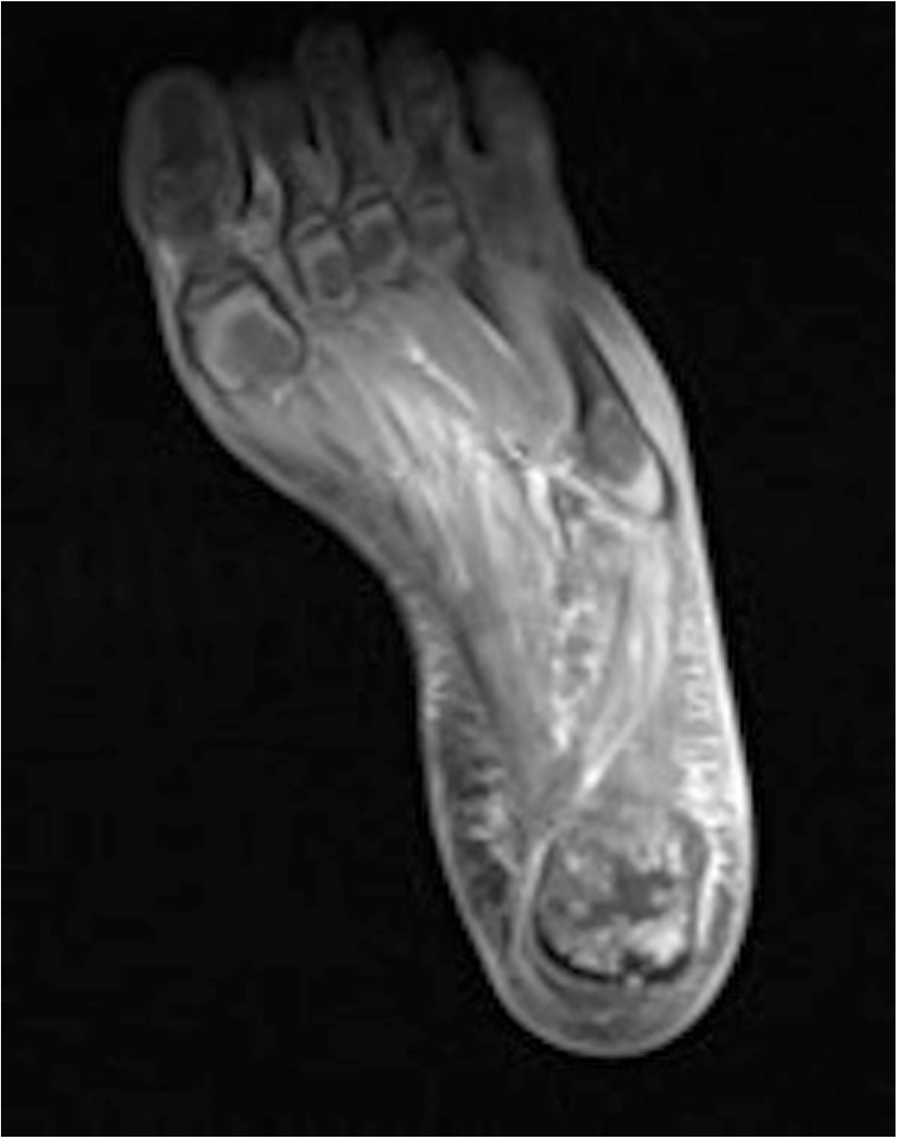
Axial magnetic resonance imaging scan of the left foot with osteomyelitis of the calcaneum

When the patient presented for a third time (a further 4 days later, and a total of 10 days after the initial onset of symptoms) it was noted that he was constitutionally unwell, pyrexial, and dehydrated with severe pain in his foot. Lymphangitis was extending up his lower leg, and the soft tissue swelling around his ankle joint was marked. Blood results revealed a white cell count (WCC) of 11.1×10^9^/L, neutrophils 8.0×10^9^/L, erythrocyte sedimentation rate (ESR) 68mm/hour and C-reactive protein (CRP) 198mg/L. An orthopedic opinion was requested, and he was promptly admitted for suspected osteomyelitis. A magnetic resonance imaging (MRI) scan of his left foot was obtained and it revealed osteomyelitis of the calcaneus bone and infection in the surrounding soft tissue (Figs. 3 and 4).

**Fig. 5:**
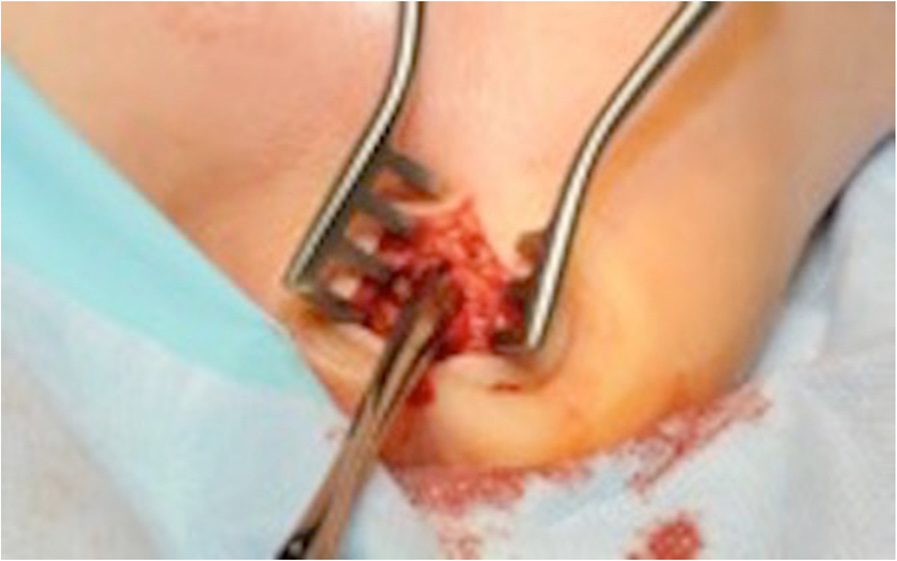
Transverse incision above os calcis to drain pus

**Fig. 6:**
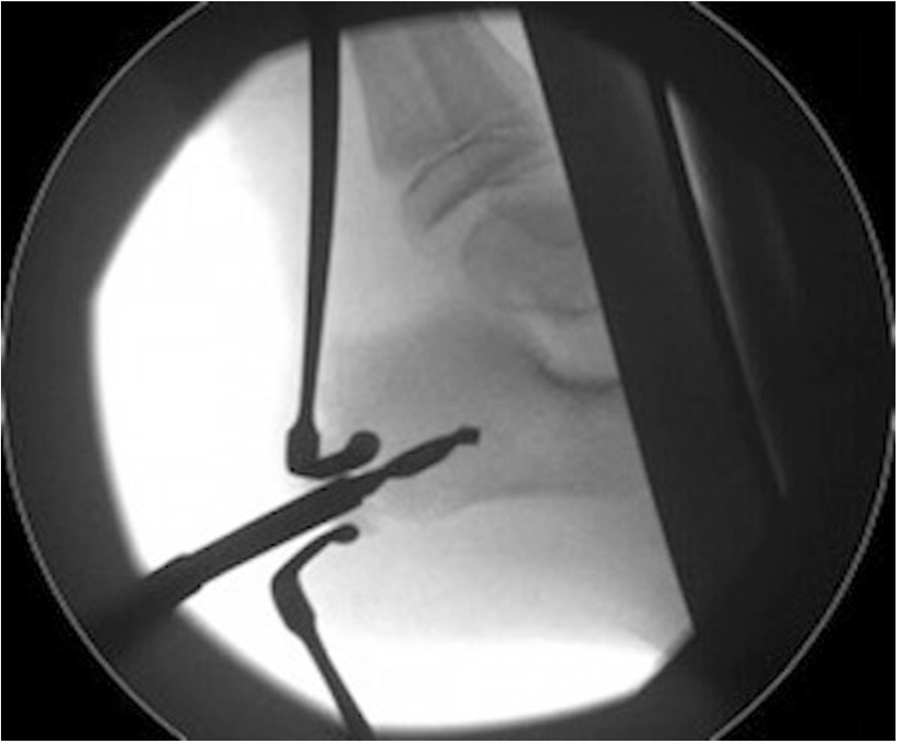
Image intensifier used to guide drilling of os calcis in theatre

After the MRI scan confirmed osteomyelitis of calcaneus, the patient was urgently taken to theatre for drainage of a left foot abscess and drilling of os calcis for osteomyelitis. A transverse incision was made and copious amounts of pus drained (sent for microscopy, culture, sensitivity, Gram stain and acid-fast culture; Fig. 5). The wound underwent a complete washout with 6 liters of saline, and the os calcis was drilled with a 4.5mm drill then washed-out further (Fig. 6). Aqueous iodine and ribbon gauze were used to gently pack the cavity and hold open the incision.

Antibiotics were withheld until after a sample was sent in theatre to ensure optimum chances of a successful culture. Penicillin-based antibiotics (flucloxacillin and co-amoxiclav) administered intravenously coupled with fusidic acid taken orally were commenced immediately after a sample was sent in theatre, as per microbiology advice. His foot was placed in a below-knee backslab to reduce movement and decrease chances of infection spread across tissue planes.

**Fig. 7:**
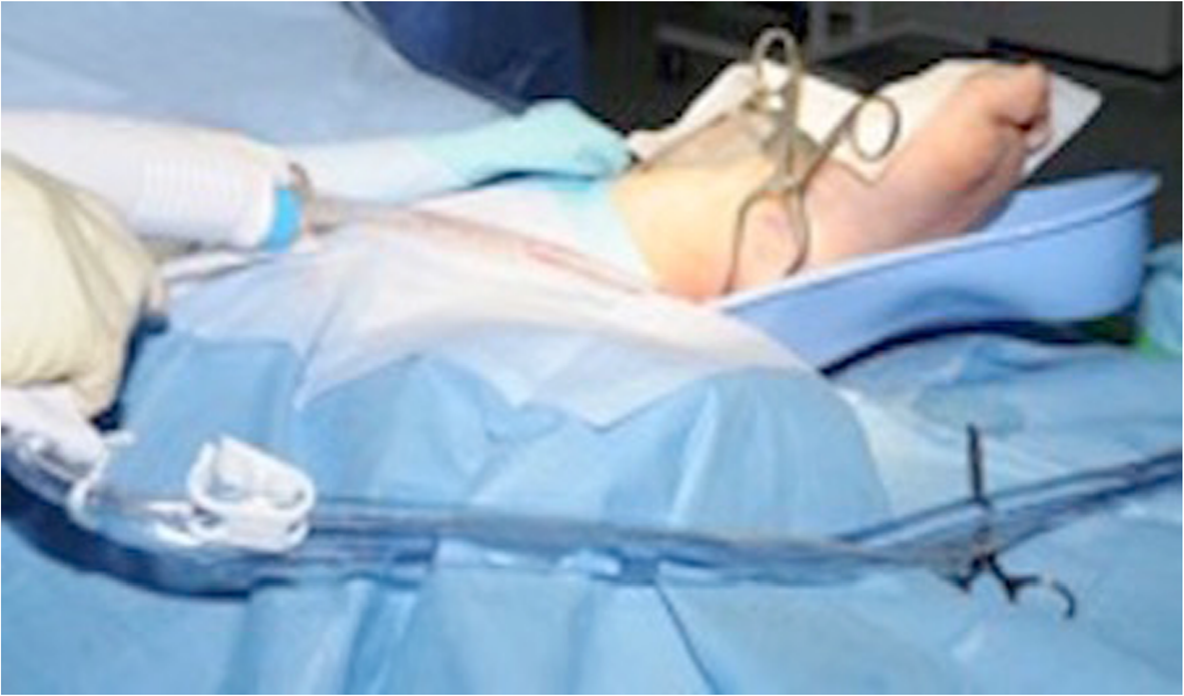
Third look wound washout with normal saline

There was regular monitoring of inflammatory markers to monitor his response to antibiotics, and two further wound washouts were performed. On the second wash (third look) the wound was healing well with granulation tissue so the wound was partially closed (Fig. 7).

*Staphylococcus aureus* was found to be responsible for the infection and, although community-acquired, it was confirmed to be a Panton-Valentine leucocidin (PVL)-negative strain. The patient continued his intravenously administered antibiotic regimen for a total of 2 weeks from the time of surgery, after which he was discharged home to continue with a 4-week course of flucloxacillin and fusidic acid administered orally. The blood results on discharge (3 weeks after admission) revealed a WCC of 5.4×10^9^/L, neutrophils 1.7×10^9^/L, ESR 18mm/hour and CRP <5mg/L.

At 6 weeks he was reviewed in clinic; his symptoms had resolved and he was able to fully weight bear without pain. We are happy to report that no complications were identified after 12 months of follow up, and he is playing football in his regional league.

## Discussion

Cases of calcaneal osteomyelitis in the pediatric population often exhibit an indolent course, with presentations less dramatic than that of long bone osteomyelitis. Delays in diagnosis and initiation of treatment are common. One study of 60 cases of calcaneal osteomyelitis by Leigh *et al*. [1] reported an average of 6.8 days before medical advice was sought, and a further 2.9 days until a diagnosis was established. Several other studies quote similar delays [2–6]. In our case there was a 10-day delay between presentation and diagnosis, with more definitive signs and symptoms developing later in the course. The diagnosis of calcaneal osteomyelitis should have been considered in this young patient presenting with heel pain at an earlier stage. Further imaging in the form of an MRI or bone scan when this patient initially presented would have clinched the diagnosis.

Calcaneal osteomyelitis can result from either direct inoculation from a puncture wound, or from hematogenous spread from distant sites. Direct inoculation may occur secondary to heel puncture for the screening of newborns [7]. Jaakkola and Kehl reported prior illnesses such as upper respiratory tract infections, otitis media and gastroenteritis in 57% of patients [4]. Therefore the respective symptoms must be sought, and trauma excluded, in a careful analysis of the history and by examination. The most common etiological agent is *Staphylococcus aureus*. Of interest, Puffinbarger *et al*. and Wang *et al*. cultured *Pseudomonas aeruginosa* in 100% of cases with puncture wounds [5, 6].

The typical signs are local tenderness, swelling, erythema and difficulty in weight bearing. Wang *et al*. [6] described a “heel up” sign, in which the child rests their ankle over the opposite knee, so that the heel does not come into contact with the bed, even whilst sleeping. Such signs and symptoms may be attributed to several conditions in childhood, such as calcaneal stress fractures, contusion and Sever’s disease. As in our case, this lengthy differential diagnosis may contribute to a delay in diagnosis.

The diagnosis of calcaneal osteomyelitis can be supported by raised WCCs and inflammatory markers. However, laboratory results are typically borderline. Elevated WCCs were found in 13 to 45% of cases quoted in the literature we reviewed [1, 3–6]. In our case the WCC was marginally elevated at 11.1×10^9^/L. Raised inflammatory markers are also somewhat variable. In our literature review, elevations in CRP on admission were reported in 22 to 77% of cases [1, 3, 4]. ESR may be a more reliable indicator, with studies quoting elevations in 81 to 95% of cases [1, 4–6]. In our case the ESR and CRP were 68mm/hour and 198mg/L respectively.

Plain radiographs can be unreliable in the early stages, with radiographic changes occurring after 7 days of onset. A retrospective study of pediatric osteomyelitis involving 156 patients showed bone scintigraphy and MRI to be the most useful imaging modalities at the onset of symptoms [8]. A study of 213 patients comparing bone scintigraphy with MRI revealed that sufficient diagnostic information was obtained in 84% of cases with scintigraphy alone. MRI scans were rarely required to obtain a diagnosis; however, 47% of cases relied on MRI to guide treatment [9]. Leigh *et al*. advocate an imaging algorithm, which begins with a plain film, and if this is normal, bone scintigraphy will be employed to localize the lesion and detect multifocality. This is followed by an MRI for improved visualization [1].

The appropriate duration of antibiotic therapy administered intravenously and the transition to antibiotics administered orally is not well defined in the literature. Winiker and Scharli quoted a mean of 9 days of parenteral antibiotics, followed by 6 weeks of oral therapy [3]. This is in contrast to Rasool, who used a longer duration of 4 to 6 weeks of parenteral antibiotics [10]. A large review of pediatric osteomyelitis literature by Dartnell *et al*. advocates a short course of antibiotics administered intravenously, followed by 3 weeks of oral therapy in uncomplicated cases of osteomyelitis. A switch to antibiotics administered orally can be made when the patient has shown signs of clinical improvement, with normalization of hematological markers. The indicators for continuing intravenous therapy are: requirement for surgical debridement, limited clinical response, methicillin-resistant *Staphylococcus aureus* (MRSA), PVL + *Staphylococcus aureus*, radiological abnormalities and failure of hematological markers to improve [11].

Traditionally, early surgical debridement and biopsy were recommended. There is little evidence for this and at present there is a trend towards medical treatment. One study of 44 patients with subacute osteomyelitis showed no difference in outcomes between medical and surgical treatment [12]. Dartnell *et al*. state that surgery should be reserved for cases of concurrent septic arthritis, disseminated sepsis and failure to improve with antibiotics [11]. Jaakkola and Kehl recommend aspiration and culture, followed by surgical debridement in cases where purulence is encountered [4]. In cases where no purulence is found, treatment with antibiotics alone is recommended in cases within 4 to 5 days of onset, with consideration of surgical debridement if symptoms do not improve with 48 to 72 hours [4]. In our case there was a delay of 10 days, and surgery was performed.

## Conclusions

Calcaneal osteomyelitis is often misdiagnosed and early diagnosis is frequently delayed. It is a challenging diagnosis due to an indolent course, blunted laboratory results, a lengthy differential diagnosis for heel pain, all in the context of frequently normal plain radiographs. It is important to initiate prompt treatment in calcaneal osteomyelitis due to the potential for growth plate disturbances, with secondary growth arrest and deformity*.* The apophysis is complete by the age of 12, and therefore the full extent of deformity may not be seen until this age. With appropriate treatment, a good outcome is likely to result.

## Consent

Written informed consent was obtained from the patient’s legal guardian for publication of this case report and any accompanying images. A copy of the written consent is available for review by the Editor-in-Chief of this journal.
